# Vasorelaxing properties of the perivascular tissue of the human radial artery

**DOI:** 10.1093/ejcts/ezac074

**Published:** 2022-02-04

**Authors:** Karolina Kociszewska, Marek Andrzej Deja, Marcin Malinowski, Adam Kowalówka

**Affiliations:** Department of Cardiac Surgery, Medical University of Silesia, School of Medicine in Katowice, Katowice, Poland

**Keywords:** Radial artery, Perivascular adipose tissue, Potassium channel blockers, CABG, Internal thoracic artery

## Abstract

**OBJECTIVES:**

Perivascular adipose tissue (PVAT) surrounding the human internal thoracic artery exhibits anticontractile and vasorelaxing properties associated with the adipocyte-derived relaxing factor (ADRF). The goal of our study was to assess if perivascular tissue of the human radial artery (RA) also exhibits such anticontractile/vasorelaxant properties. It could be especially relevant in preventing RA spasms.

**METHODS:**

The study was performed on isolated segments of human pedicled RA. Its skeletonized fragments were suspended on stainless steel wire hooks and gradually contracted with serotonin to establish the concentration–effect relationship in the presence/absence of PVAT. Skeletonized arterial segments were precontracted with a single dose of 10^−6^ M serotonin (EC_80_). The 5-ml PVAT aliquots (from PVAT incubated in Krebs–Henseleit solution) were transferred to the RA tissue bath resulting in its relaxation. Subsequently, we investigated if ADRF is dependent on endothelial vasorelaxants (nitric oxide and prostacyclin). We attempted to find the potassium channel responsible for mediating the activity of ADRF using different potassium channel blockers.

**RESULTS:**

RA without PVAT contracted more strongly in response to serotonin compared to RA with PVAT [*E*_max_: 108.3 (20.2) vs 76.1 (13.5) mN]. The PVAT aliquot relaxed precontracted RA rings at 43% (2.4%) [72.2 (15.6) to 41.0 (5.6) mN]. ADRF is independent of endothelial vasorelaxants; hence, the addition of NG-monomethyl-l-arginine and indomethacin did not change the vasorelaxant response. Neither of the potassium channel blockers participated in the activity of ADRF.

**CONCLUSIONS:**

PVAT of human RA exhibits anticontractile/vasorelaxant properties that are inherently associated with ADRF secretion. We confirmed the endothelial-independent mechanism of the activity of ADRF. However, we failed to find the potassium channel responsible for the action of ADRF.

**Figure:**
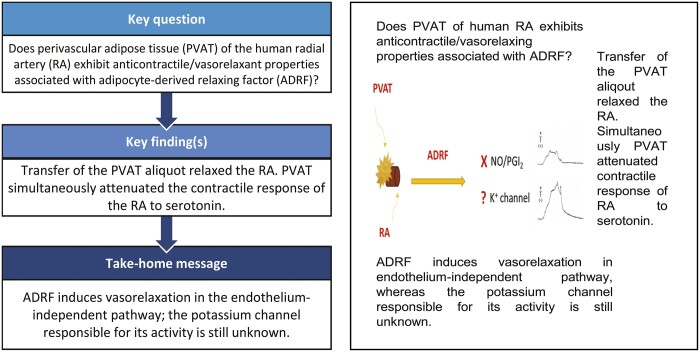

## INTRODUCTION

In 1973, Carpentier indicated the radial artery (RA) as a graft of second choice in coronary artery bypass grafting [1]. Many studies, like the Radial Artery Patency Study or the Radial Artery Patency and Clinical Outcomes trial, confirmed its benefits, like long-term patency [2]. Some of the unique morphological properties of the RA together with the endo- and paracrine activities of its endothelium [capable of the release of nitric oxide (NO) and prostaglandin I2 (prostacyclin)] determine the superiority of the RA graft in comparison to the saphenous vein [3, 4]. Still, many surgeons feel that, due to its specific muscular nature, it is susceptible to spasm, which in turn could promote early graft failure [5, 6]. Indeed, thick, muscular media together with hyperplasia of intimal cells may easily endanger the patency of the small-lumen RA graft [5]. On the other hand, the preserved reactivity of the arterial conduit may be beneficial in the long run by adjusting the blood flow according to need and preventing blood stasis within the conduit.

Our previous studies confirmed that the perivascular tissue of the human internal thoracic artery (ITA) exerts an anticontractile effect on the underlying vascular wall and that this effect is associated with the release of adventitia or adipocyte-derived relaxing factor (ADRF)/perivascular tissue-derived relaxing factor (PVRF) [7, 8]. ADRF, first described by Soltis and Cassis in 1991, attenuates the constrictive vascular response to plenty of vasoactive agents, independently of the endothelium and NO or prostacyclin release [4, 7, 9]. It acts by activating the potassium channels: K_v_ in the mesenteric arteries of rats and mice; KCNQ delayed-rectifier Ca2+-sensitive large-conductance potassium channel (K_Ca_) in the aortas of rats and mice; and the adenosine triphosphate-dependent potassium channel (K_ATP_) in humans, where it potentially participates in ADRF-mediated relaxation [10, 11]. We concluded that harvesting the ITA as a pedicle might therefore be beneficial [7, 8]. Whether perivascular fat surrounding the RA is also a source of ADRF remains unclear. The answer to this question might be especially important because, with the advent of the endoscopic technique, RA is more and more often harvested skeletonized. If the perivascular adipose tissue (PVAT) surrounding the RA exhibits anticontractile/vasorelaxing properties, preserving the RA pedicle could help to prevent postoperative vasospasm and potentially improve its physiological properties as a coronary artery graft.

Thus, our study was designed to identify the anticontractile/vasorelaxing properties of PVAT surrounding the human RA and determine if those properties are associated with a transferable vasorelaxing factor. We also attempted to determine the mechanism of ADRF/PVRF.

## MATERIALS AND METHODS

The study lasted from October 2016 until April 2020. The experiments were performed in an isolated organ laboratory belonging to the Department of Cardiac Surgery in Katowice, Poland. The study was performed on isolated segments of human RA discarded during coronary artery bypass grafting for stable coronary artery disease after the conduit had been trimmed to the length necessary for revascularization.

The exclusion criteria were age >70 years, concomitant diseases at an advanced stage and a concomitant cardiac procedure. The inclusion and exclusion criteria were similar across the different experiments; however, in every experiment, we used material from different patients.

### Ethics statement

The local bioethics committee (Bioethics Committee of the Medical University of Silesia, Katowice, Poland) agreed to the study and granted the waiver for the use of this waste tissue without obtaining patients’ informed consent (approval number: KNW/0022/KB/186/16). The recruitment was not consecutive. All RA grafts were harvested pedicled using an open surgical technique. The discarded fragments of RA were placed in cold (4°C) calcium-free modified Krebs–Henseleit solution (123 mM NaCl, 4.7 mM KCl, 1.64 mM MgSO_4_, 24.88 mM NaHCO_3_, 1.18 mM KH2PO_4_, 5.55 mM glucose, 2 mM sodium pyruvate, pH 7.4). They were transferred immediately to the laboratory and dissected free of the surrounding PVAT. Next, they were divided into 3-mm-long rings and suspended on stainless steel wire hooks in the 20-ml organ bath chamber filled with oxygenated (95% O_2_, 5% CO_2_) Krebs–Henseleit solution (119.0 mM NaCl; 4.70 mM KCl; 1.6 mM CaCl_2_; 1.2 mM MgSO_4_; 25.0 mM NaHCO_3_; 1.2 mM KH_2_PO_4_; 11.01 mM glucose; 2.0 mM sodium pyruvate 2.0; pH 7.4). We maintained a temperature of 37°C. The Schuler isolated organ bath (Hugo Sachs Elektronik; March-Hugstetten, Germany) was used. Vessel wall tension was measured with the isometric force transducer F 30 (Hugo Sachs Elektronik); the signal was enhanced with bridge amplifier type 336 (Hugo Sachs Elektronik) and recorded using the PowerLab/4SP system and Chart software (AD Instruments, Chalgrove, Oxfordshire, UK). After 15 min of initial incubation, the standardized procedure described by Mulvany and Halpern [12] was applied to normalize vessel wall tension and diameter. It set every vessel to 90% of the diameter it would have had *in vivo* when relaxed and under the transmural pressure of 100 mmHg using Laplace’s law: *P* = 2*T*/*d*. Subsequently, the vessel was left to stabilize for 30 min. To check for the viability (contractile response) of the artery, we applied 60 mM KCl with subsequent washout before starting the experiment. To confirm endothelial integrity, 10^−5^ M acetylcholine was used at the end of every experiment.

### Experiment 1

**Figure 1:**
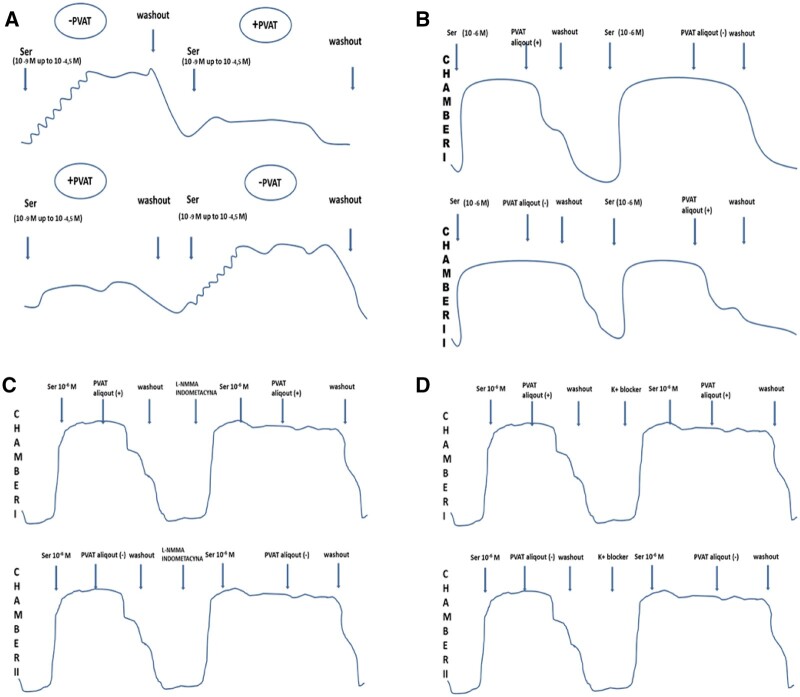
(**A**) One segment was incubated with perivascular adipose tissue and the other without perivascular adipose tissue. First, both arterial rings were gradually contracted with serotonin starting from 10^−9^ M and rising in negative logarithm half-molar cumulative steps up to 10^−4.5^ M to establish the concentration-effect relationship in the presence/absence of perivascular adipose tissue. After washout and transfer of loose perivascular adipose tissue, the concentration-response curves with the same contractile factor were reconstructed. (**B**) Both arterial rings were precontracted with a single dose of 10^−6^ M serotonin. The perivascular adipose tissue tissue was floating freely in the solution during the experiment, presumably releasing adipocyte-derived relaxing factor into the bath. Having achieved a plateau of the contraction to serotonin, the 5-ml perivascular adipose tissue aliquot was added to 1 arterial segment with another one serving as a control. Various levels of relaxation were observed after adding the perivascular adipose tissue aliquot. (**C**) After washout and a stabilization period, we added NG-monomethyl-l-arginine to the first 5 specimens and indomethacin to the next 5 specimens. After incubation, we observed the contraction of 1 arterial segment in response to a single, submaximal dose of serotonin and then some relaxation associated with the perivascular adipose tissue aliquot. (**D**) After washout and the stabilization period, we added potassium channel blockers. After incubation, we observed the contraction of 1 arterial segment with a single, submaximal dose of serotonin and then some relaxation/contraction associated with the 5-ml perivascular adipose tissue aliquot. INDO: indomethacin; K+ blocker: potassium channel blocker; l-NMMA: NG-monomethyl-l-arginine; PVAT: perivascular adipose tissue; PVAT aliquot: Krebs–Henseleit solution with incubated PVAT; Ser: serotonine.

The first part of the bioassay was designed to prove the anticontractile properties of RA PVAT. Thus, we compared the reactivity of the RA to serotonin in the presence or absence of perivascular tissue obtained from 15 patients. The RA fragment was divided into two 3-mm skeletonized segments. Both segments were studied simultaneously, 1 with perivascular tissue floating freely in the bath (PVAT+) and the other alone, without PVAT (PVAT−). The mean weight of the PVAT used in the experiment was 573 ± 360 mg. Both arterial rings were simultaneously gradually contracted with serotonin starting from 10^−9^ M and rising in negative logarithm half molar cumulative steps up to 10^−4.5^ M to establish the concentration–effect relationship in the presence/absence of PVAT (Fig. 1A). Then, after washout, PVAT was transferred from 1 bath to another, and the concentration-response curves to serotonin were reconstructed. This way we obtained 2 consecutive concentration-response curves in each RA ring while switching the order of the (PVAT+/−) conditions.

### Experiment 2

The second part of the bioassay was designed to assess the vasorelaxing properties of human RA PVAT. However, this time we transferred the PVAT aliquot instead of the perivascular fat. RA segments, obtained from 12 patients, were each divided into 2 skeletonized 3-mm rings and were suspended on 2 stainless steel wire hooks. The PVAT remaining from skeletonization was incubated separately in another organ bath chamber under the same conditions in the Krebs–Henseleit solution for at least 1 h. This way we obtained the PVAT aliquot. The mean weight of PVAT was 727 ± 293 mg. After equilibration, stabilization and normalization, both preparations were precontracted with a single dose of 10^−6^ M serotonin at a concentration equivalent to the submaximally effective (EC_80_) concentration in experiment 1. Serotonin was added also to the chamber with freely floating PVAT to maintain the concentration of the vasoconstrictor in the PVAT aliquots equal to that in the arterial ring bath.

The 5-ml PVAT aliquot was transferred to 1 of the serotonin precontracted arterial segments with another one serving as a control. Next, both arterial segments were washed and precontracted again, and the PVAT aliquot was added to the segment that previously served as a control (Fig. 1B).

### Experiment 3

The primary part of the third experimental protocol is the same as that in the second experiment. We obtained the RA rings from 10 patients. The mean weight of PVAT used in the experiment was 622 ± 285 mg. Thus, the RA was divided into 2 skeletonized segments while the remaining PVAT was floating freely in the separated bath chamber. After preparation, both arterial segments were precontracted with a single, submaximal dose of serotonin—10^−6^ M. By analogy, the same dose of serotonin was added to the chamber with PVAT. After the plateau phase of contraction was achieved, the 5-ml PVAT aliquot was transferred to 1 of the serotonin precontracted arterial segments with another one serving as a control. After the washout and stabilization period, we added NG-monomethyl-l-arginine (10^−4^ M) to the first 5 specimens and indomethacin (10^−5^ M) to the next 5 specimens. After ∼30 min of incubation, we added a submaximal dose of serotonin and subsequently a 5-ml aliquot of PVAT (Fig. 1C).

### Experiment 4

The first phase of the experimental protocol was the same as in the 3rd experiment. We obtained RA rings from 56 patients. However, this time, instead of endothelial vasorelaxants blockers, we added potassium channels blockers, such as:

1. BaCl_2_ = 100 μM (416.5 μg) (*n* = 8)—inward rectifier potassium channel (K_IR_) blocker
2. Tetraethylammonium = 1 mM (3.3142 mg) (*n* = 8)—non-selective BK_Ca_ blocker
3. Apamine = 1 μM (40.54 μg) (*n* = 8) – SK_Ca_ blocker
4. 4-Aminopyridine = 1 mM (1.8824 mg) (*n* = 8) and 5 mM (9.412 mg) (*n* = 8)—K_v_ blocker
5. Iberiotoxin = 100 nM (8.46 μg) (*n* = 8)—selective BK_Ca_ blocker
6. Glibenclamide = 10 μM **(**988 μg) (*n* = 8) and 5 μM (494 μg) (*n* = 4)—K_ATP_ blocker.

The mean weight of PVAT used in the experiment was 711 ± 308 mg. After a 30-min incubation period, we added a single, submaximal dose of serotonin and subsequently a 5-ml aliquot of PVAT (Fig. 1D).

### Statistical analyses

Vessel wall tension was measured and expressed in mN (millinewton). The artery contraction was measured as an increase in the vessel wall tension above the resting tension. The relaxation was assessed as the decrease in wall tension from the precontracted level and expressed as a percentage of the contraction obtained with serotonin. The perivascular tissue weight is presented as the mean with standard deviation.

We present the contraction responses as mean ± SEM and compare them at each concentration level using the paired *t*-test. The concentration–effect relationship is estimated with regression analysis using the general logistic equation of Hill and Langmuir:

$$E=\frac{E_{\max}\times D^{n}}{D^{n}+K_{D}^{n}}$$

where *E* is the effect, *E*_max_ is the maximal effect, *D* is the concentration, KD is the drug–receptor complex dissociation constant equal to the concentration causing half-maximal effect (EC_50_) and *n* is the Hill coefficient. Data from 2 concentration-response curves obtained in the presence of PVAT were pooled into 1 regression analysis. A separate regression analysis was performed for data obtained in the absence of PVAT. The parameters of the regression analysis within every experiment were compared using the Student's *t*-test.

In the third and fourth experiments, the parameters of relaxation in both the control and the study groups were compared using the Student's *t*-test and two-way repeated measures of analysis of variance (ANOVA)—two-factor repetition, where the PVAT aliquot was 1 factor and the presence of an adequate inhibitor was another factor.

In all of the statistical analyses, *P* < 0.05 was considered significant. All analyses were performed using SigmaPlot 12.5 and Sigma Stat 3.5 Software (Systat, Inc., San Jose, CA, USA).

## RESULTS

### Experiment 1

**Figure 2:**
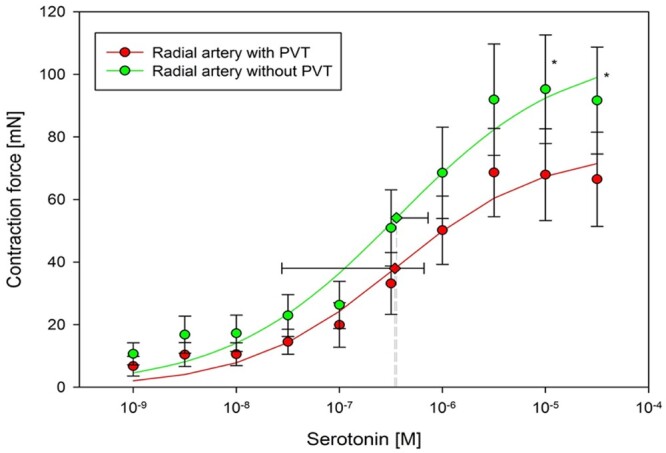
The presence of perivascular adipose tissue in the bath caused a significantly lower maximal response to serotonin compared to the preparation without perivascular adipose tissue. Removing perivascular adipose tissue resulted in the augmentation of the contraction response to the vasopressor in the donor vessel, whereas the transfer induced the decrease of *E*_max_ in the recipient preparation. The concentration-response relationship pooled from 2 curves showed that the presence of perivascular adipose tissue in the bath caused decrease of *E*_max_ in response to serotonin (*E*_max_: 108.3 ± 20.2 vs 76.1 ± 13.5 mN). PVT = PVAT: perivascular adipose tissue.

If perivascular tissue had secreted ADRF into the bath, the contractile response of the vessels would have been affected. PVAT floating in the tissue bath resulted in a significantly lower maximal response to serotonin compared to the preparation without PVAT (Fig. 2). PVAT removal resulted in an increase of contraction in response to the vasopressor in the donor’s vessel, whereas the transfer induced a decrease of *E*_max_ in the recipient preparation. The estimated maximal contraction in response to serotonin was 108.3 (20.2) mN and decreased to 76.1 (13.5) mN by the presence of PVAT in the tissue bath (*P* < 0.0001; *n* = 15). The EC_50_ for serotonin was not changed by the presence or absence of PVAT in the solution (3.57 × 10^−7^ ± 3.70 × 10^−7^ M vs 3.45 × 10^−7^ ± 3.18 × 10^−7^ M; *P* = 0.3).

### Experiment 2

**Figure 3:**
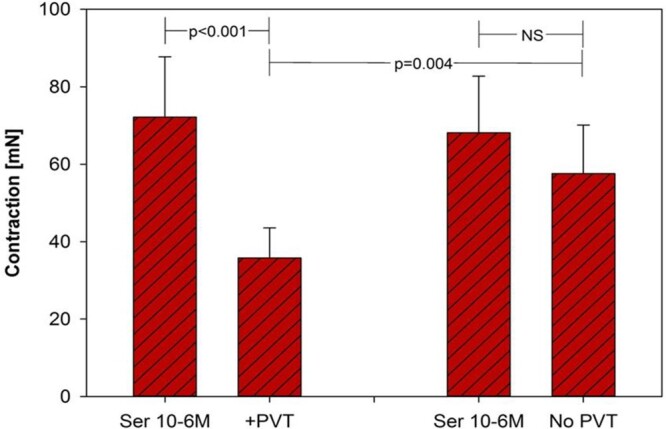
Serotonin elicited a contraction of the radial artery of 72.2 ± 15.6 mN. The addition of 5 ml of the perivascular adipose tissue aliquot to the skeletonized radial artery induced approximately a 43% decrease in *E*_max_ in response to serotonin (72.2 ± 15.6 to 41.0 ± 5.6 mN). No significant relaxion was observed in the control arterial segment (68.1 ± 14.7 vs 66.6 ± 9.9 mN). PVT = PVAT: perivascular adipose tissue; Ser: serotonin.

Serotonin (10^−6^ M) elicited a contraction of the RA [72.2 (15.6) mN]. The addition of 5 ml of PVAT-treated solution relaxed RA to 41.0 (5.6) mN (*P* < 0.001; *n* = 24). No significant relaxation was observed in the control arterial segment [68.1 (14.7) vs 65.6 (9.9) mN, *P* = 0.4; *n* = 24] (Fig. 3).

### Experiment 3

Both NG-monomethyl-l-arginine and indomethacin had no impact on perivascular tissue anticontractile properties (Table 1).

**Table 1:** Influence of nitric oxide and prostacyclin inhibitors influence on the anticontractile properties of perivascular adipose tissue

	l-NMMA (−)	l-NMMA (+)	*P*-Value
PVAT (+)	20 ± 2.9%	31 ± 11.8%	l-NMMA (−) versus l-NMMA (+) = 0.151
PVAT (−)	−21 ± 9.2%	6 ± 11.9%	
*P*-Value	PVAT (+) versus PVAT (−) = 0.005		PVAT (+) × l-NMMA (+) = 0.223
	INDO (−)	INDO (+)	*P*-Value
PVAT (+)	23 ± 8.3%	15 ± 3.8%	INDO (−) versus INDO (+) = 0.167
PVAT (−)	15 ± 5.4%	4 ± 2.6%	
*P*-Value	PVAT (+) versus PVAT (−) = 0.006		PVAT (+) × INDO (+) = 0.545

INDO: indomethacin; l-NMMA: NG-monomethyl-l-arginine; PVAT: perivascular adipose tissue.

### Experiment 4

Two-way repeated measures of ANOVA showed a significant impact of the PVAT aliquot on vessel relaxation; however, the test did not confirm the influence of any potassium channel blocker on the relaxation induced by the PVAT aliquot. ANOVA merely confirmed the significant impact of barium chloride on the vessel wall tension.

The results are presented as tables in the Supplementary Material.

## DISCUSSION

Our study confirmed for the first time that the perivascular tissue of the human RA exhibits anticontractile/vasorelaxant properties. Transfer of the Krebs–Henseleit solution treated with PVAT into the organ chamber containing the skeletonized RA resulted in its relaxation. At the same time, PVAT attenuated the contractile response of RA to serotonin. Because the effect is transferable with a PVAT-treated solution, it seems likely that this effect is mediated by ADRF released from the perivascular tissue surrounding the RA, as is the case with the human ITA.

The RA is much more difficult to examine and more demanding in comparison to the human ITA. It requires a longer period of normalization or stabilization, and it often escapes outside the frames of predicted activity. Because its capacity to strong contraction exceeded the scale, more than once we were prevented from completing the experiment. Because the intensity of both contraction and relaxation is different in every specimen, we decided to take into account the relative level of relaxation. We compared specific contraction and relaxation levels between the study and the control groups in a specific specimen. Hence, we obtained diverse levels of dilation, fluctuating between 11% and 43%. Actually, a few factors should be considered as the reasons for this discrepancy, such as the different clinical state and pharmacotherapy of each patient, the various histological structures (domination of white adipose tissue or brown adipose tissue) or even the different weights of the perivascular tissue.

In the first experiment, we proved that PVAT exhibits anticontractile activity even without continuity with the vascular wall. Indeed, PVAT remained after skeletonization was floating freely in the organ bath chamber with a denuded arterial ring, presumably releasing ADRF/PVRF into the bath.

In the second experiment, we showed that ADRF/PVRF not only acts in the presence of PVAT but is being released into the bath chamber during incubation. Moreover, it could be transferred to another bath chamber in the aliquot without loss of vasorelaxing/anticontractile properties.

Simultaneously, we should bear in mind that when we add the PVAT aliquot or the PVAT itself floats freely in the bath separated from the vascular wall, ADRF/PVRF acts on both sides of the arterial ring—intraluminally or externally. The vast majority of studies analysed ADRF secretion in physiological conditions with unseparated PVAT [10, 13–16]. We did not observe a particular difference in the intensity of contraction for serotonin independently from PVAT floating freely or the addition of a PVAT aliquot. Thus, our bioassay revealed, in part, the nature of ADRF, which could act with or without continuity with PVAT.

We confirmed that ADRF/PVRF acts independently of NO and prostacyclin. The anticontractile effect was still clear after the application of NO synthase and cyclooxygenase inhibitors, which excluded the participation of 2 basic vasorelaxants, as in the endothelium [4, 15]. It contradicted Gao’s claim that the anticontractile properties of PVAT result from endothelial activity [17].

We failed to confirm the participation of potassium channels in the activity of ADRF in human RA. Initially, K_IR_ appeared to be the responsible channel; however, the analysis of the control group excluded such a possibility. The increase in contraction in response to serotonin after the application of BaCl_2_ was apparent in both groups.

The only channel left untested was K_ATP_. Despite numerous attempts and changes in glibenclamide dosage, we could not obtain reliable data. Thus, it could be the mediator of the activity of ADRF. However, this channel is associated with metabolic processes, not with vascular wall regulation The participation of BK_Ca_ channels seems to be the most probable explanation, because their role in vasorelaxation is widely described and accepted. Still, we excluded this possibility. Even if those channels mediate anticontractile activity in human RA, for certain they are not dominant. On the other hand, our observations from the experiment with BaCl_2_, indicate that K_IR_ channels are the important mediator in the maintenance of basic vascular wall tension. We directly proved that those channels could play a role in vascular wall regulation and in the balance between contraction and relaxation.

Most of the current research concentrates on the role of ADRF in animal vascular beds: the rat mesenteric bed or the mouse aorta [7–10, 15]. These studies triggered the search for the possible vasorelaxing role of perivascular tissue in humans. Human studies involved mainly perivascular tissue of the internal thoracic artery [7, 11, 18]. We believe that the knowledge of perivascular tissue and the role of ADRF in vascular tone modulation in the animal aorta, mesenteric bed or human ITA did not allow for a simple generalization of the roles of PVAT and ADRF in the RA [4].

Our study excluded the role of potassium channels as the important mediators of ADRF activity. It seems that vasorelaxant mechanisms might differ among particular levels and systems of the vascular bed. In human ITA and in studied animal vascular beds, ADRF indeed acts through potassium channels. In human RA, the situation seems much more difficult. Hence, it is possible that in different parts of the vascular bed, there are various potassium channels and the distribution or density of vasorelaxant receptors might differ. For instance, in 1 part of the vascular bed, the major determinant could be „shear stress” in other widely described BK_Ca_ channels. Also, purinergic receptors like P2Y1 should be further investigated. Moreover, the factor that relaxes 1 vascular bed may not act the same way in other vascular beds. Different types of adipose tissues in diverse anatomical regions could have different vasorelaxant properties and release different substances. The experiment with the cross-reaction between PVAT of ITA and RA could provide a partial answer. Further biochemical studies using mass spectrometry could help in discovering the chemical nature of ADRF.

The different susceptibilities to spasm between RA and ITA could be explained by diverse BK_Ca_ expression [19]. In the study by Shao *et al.* [19], tetraethylammonium and iberiotoxin (BK_Ca_ channel blockers) had significantly stronger inhibiting effects than relaxing effects on human ITA in comparison to the effect on RA, whereas the expression of those channels was twice as high in the muscular layer of ITA as it was in the RA.

Our study results augment the hypothesis that perivascular tissue and ADRF are indeed important players in the physiology of vascular tone regulation in humans [20]. Our hypothesis is further supported by the fact that, in some pathological states, including obesity, arterial hypertension or diabetes, the function of ADRF is impaired [21, 22]. Also, there might be a limit to the beneficial role of perivascular tissue. Indeed, in patients with obesity or hypertension, the beneficial action of PVAT and ADRF on the human ITA could be completely abolished [21, 22]. Perivascular tissue becomes a source of vasoconstrictive agents, the location of an inflammatory response, implicating impaired vascular behaviour. Those effects are not dependent on the amount of PVAT. On the one hand, a decreased amount of PVAT is observed in some animal models of hypertension, yet its higher content in obesity is associated with loss of the anticontractile effect [21, 22].

### Limitations

We obtained RA specimens from a diverse group of patients with a whole range of disease states and pharmacotherapeutic regimens; thus different factors might affect vascular function as well as our results and conclusions.

Tachyphylaxis and carry-over effects may also limit the reliability of our study.

Moreover, our sample size could be too small to detect the results we were looking for. Amplification of the sample size would allow us to perform multifactorial analyses and increase confidence in our results.

The study is an *in vitro* isolated bath study, deprived of constant blood flow, tissue perfusion, oxygen delivery and the elimination of metabolites. The discrepancy between the results of our study and those previously described could stem from the differences between the species or the experimental designs.

## CONCLUSIONS

We proved that the perivascular tissue surrounding the human RA exhibits anticontractile/vasorelaxing properties, associated in all likelihood with ADRF secretion. Moreover, the release of ADRF is independent of the endothelium. We did not confirm the participation of potassium channels as significant mediators of ADRF activity. It could be explained in part by the theory of different paracrine agents and receptors among the species and the vascular beds. It seems, therefore, that harvesting the RA conduit as a pedicle could be more beneficial than harvesting it skeletonized. Further work is needed to discover the mechanisms through which this phenomenon occurs. The major goal in harvesting RA is to obtain and anastomose the vessel without a spasm. Thus, the preservation of PVAT could be yet another way to protect the artery against an exaggerated contractile response and may add to the topical or systemic use of vasodilators.

## SUPPLEMENTARY MATERIAL

Supplementary material is available at *EJCTS* online.

## Supplementary Material

ezac074_supplementary_data

## ABBREVIATIONS

ADRF: Adipocyte-derived relaxing factor
ANOVA: Analysis of variance
ITA: Internal thoracic artery
K_ATP_: Adenosine triphosphate-dependent potassium channel
K_Ca_: KCNQ delayed-rectifier Ca2+-sensitive large-conductance potassium channel
K_IR_: Inward recitifier potassium channel
NO: Nitric oxide
PVAT: Perivascular adipose tissue
PVRF: Perivascular tissue-derived relaxing factor
RA: Radial artery

## References

[1] Petrovic I , Nezic D, Peric M, Milojevic P, Djokic O, Kosevic D et al Radial artery vs saphenous vein graft used as the second conduit for surgical myocardial revascularization: long-term clinical follow-up. J Cardiothorac Surg 2015;10:127. 26466996 10.1186/s13019-015-0331-9 PMC4606847

[2] Tranbaugh RF , Dimitrova KR, Friedmann P, Geller CM, Harris LJ, Stelzer P et al Coronary artery bypass grafting using the radial artery: clinical outcomes, patency, and need for reintervention. Circulation 2012;126:S170–5. 22965979 10.1161/CIRCULATIONAHA.111.083048

[3] Lüscher TF , Diederich D, Siebenmann R, Lehmann K, Stulz P, von Segesser L et al Difference between endothelium-dependent relaxation in arterial and in venous coronary bypass grafts. N Engl J Med 1988;319:462–7. 3136329 10.1056/NEJM198808253190802

[4] Malinowski M , Deja MA, Gołba KS, Roleder T, Biernat J, Woś S. Perivascular tissue of internal thoracic artery releases potent nitric oxide and prostacyclin-independent anticontractile factor. Eur J Cardiothorac Surg 2008;33:225–31. 18083040 10.1016/j.ejcts.2007.11.007

[5] Fleissner F , Engelke H, Rojas-Hernandez S, Ismail I, Stiefel P, Cebotari S et al Long-term follow-up of total arterial revascularization with left internal thoracic artery and radial artery T-grafts: survival, cardiac morbidity and quality of life. Eur J Cardiothorac Surg 2016;49:1195–200. 26377637 10.1093/ejcts/ezv289

[6] Rudzinski P , Wegrzyn P, Lis GJ, Piatek J, Konstanty-Kalandyk J, Nosalski R et al Vasodilatory effect and endothelial integrity in papaverine- and milrinone-treated human radial arteries. J Physiol Pharmacol 2013;64:41–5. 23568970

[7] Kociszewska K , Malinowski M, Czekaj P, Deja MA. What is the source of anticontractile factor released by the pedicle of human internal thoracic artery? Interact CardioVasc Thorac Surg 2015 ;21:301–7. 26082494 10.1093/icvts/ivv142

[8] Deja MA , Gołba KS, Malinowski M, Woś S, Kolowca M, Biernat J et al Skeletonization of internal thoracic artery affects its innervation and reactivity. Eur J Cardiothorac Surg 2005 ;28:551–7. 16125945 10.1016/j.ejcts.2005.06.037

[9] Soltis EE , Cassis LA. Influence of perivascular adipose tissue on rat aortic smooth muscle responsiveness. Clin Exp Hypertens A 1991;13:277–96. 2065467 10.3109/10641969109042063

[10] Dubrovska G , Verlohren S, Luft FC, Gollasch M. Mechanisms of ADRF release from rat aortic adventitial adipose tissue. Am J Physiol Heart Circ Physiol 2004;286:H1107–13. 14644761 10.1152/ajpheart.00656.2003

[11] Malinowski M , Deja MA, Janusiewicz P, Golba KS, Roleder T, Wos S. Mechanisms of vasodilatatory effect of perivascular tissue of human internal thoracic artery. J Physiol Pharmacol 2013;64:309–16. 23959727

[12] Mulvany MJ , Halpern W. Contractile properties of small arterial resistance vessels in spontaneously hypertensive and normotensive rats. Circ Res 1977;41:19–26. 862138 10.1161/01.res.41.1.19

[13] Verlohren S , Dubrovska G, Tsang SY, Essin K, Luft FC, Huang Y et al Visceral periadventitial adipose tissue regulates arterial tone of mesenteric arteries. Hypertension 2004;44:271–6. 15302842 10.1161/01.HYP.0000140058.28994.ec

[14] Gollasch M , Dubrovska G. Paracrine role for periadventitial adipose tissue in the regulation of arterial tone. Trends Pharmacol Sci 2004;25:647–53. 15530643 10.1016/j.tips.2004.10.005

[15] Löhn M , Dubrovska G, Lauterbach B, Luft FC, Gollasch M, Sharma AM. Periadventitial fat releases a vascular relaxing factor. FASEB J 2002;16:1057–63. 12087067 10.1096/fj.02-0024com

[16] Wang N , Kuczmanski A, Dubrovska G, Gollasch M. Palmitic acid methyl ester and its relation to control of tone of human visceral arteries and rat aortas by perivascular adipose tissue. Front Physiol 2018;9:583. 29875688 10.3389/fphys.2018.00583 PMC5974537

[17] Gao YJ , Lu C, Su LY, Sharma AM, Lee RM. Modulation of vascular function by perivascular adipose tissue: the role of endothelium and hydrogen peroxide. Br J Pharmacol 2007;151:323–31. 17384669 10.1038/sj.bjp.0707228 PMC2013985

[18] Deja MA , Malinowski M, Golba KS, Piekarska M, Wos S. Perivascular tissue mediated relaxation - a novel player in human vascular tone regulation. J Physiol Pharmacol 2015;66:841–6. 26769833

[19] Shao Y , Liew R, Guan B, Wong PE, Shim WS, Chua Y et al Different expression of large-conductance calcium-activated K+ channels in human internal mammary and radial arteries. Cardiovasc Res 2011;89:329–35. 20870652 10.1093/cvr/cvq304

[20] Tano JY , Schleifenbaum J, Gollasch M. Perivascular adipose tissue, potassium channels, and vascular dysfunction. Arterioscler Thromb Vasc Biol 2014;34:1827–30. 25012133 10.1161/ATVBAHA.114.303032

[21] Fernández-Alfonso MS , Gil-Ortega M, García-Prieto CF, Aranguez I, Ruiz-Gayo M, Somoza B. Mechanisms of perivascular adipose tissue dysfunction in obesity. Int J Endocrinol 2013;2013:402053. 24307898 10.1155/2013/402053 PMC3838835

[22] Szasz T , Webb RC. Perivascular adipose tissue: more than just structural support. Clin Sci 2012;122:1–12. 10.1042/CS20110151 PMC3966487 21910690

