# Supplementary material for: Vasorelaxing properties of the perivascular tissue of the human radial artery
Source: Eur J Cardiothorac Surg. 2022 Feb 4;61(6):1423–9. doi: 10.1093/ejcts/ezac074 (PMC9728790; doi:10.1093/ejcts/ezac074)
Supplement: ezac074_supplementary_data [file ezac074_supplementary_data.docx]

**SUPPLEMENTARY DATA:**

**Experiment 3:**

1. L-NMMA:

n=5

|  | **L-NMMA (-)** | **L-NMMA (+)** | **p** |
| --- | --- | --- | --- |
| **PVAT (+)** | 20 ± 2,9% | 31 ± 11,8% | L-NMMA (-) vs L-NMMA (+)=0,151 |
| **PVAT (-)** | -21 ± 9,2% | 6 ± 11,9% |  |
| **p** | PVAT (+) vs PVAT (-) = 0,005 | | PVAT (+)*L-NMMA (+)=0,223 |

The serotonin elicited a contraction of the RA 31,7±4,8mN in the study group vs 19,7±6,8mN in the control group. The addition of serotonin in the presence of L-NMMA induced higher contractory response in comparison to specimens subjected to serotonin itself (increase from 31,7±4,8mN to 49,6±8,6mN, p=0,004 in the study group; increase from 19,7±6,8mN to 38,6±13,2mN vs, p=0,009 in the control group). Thus, nitric oxide synthase inhibitor augmented contractory response to serotonin.

The addition of 5 ml of PVAT-treated solution relaxed RA by 20% in the study group. While the relaxation in the control group treated with Krebs-Henseleit solution instead of PVAT aliquot wasn’t observed, even it contracted the artery by 21%.

The addition of 5 ml of PVAT-treated solution in the presence of L-NMMA relaxed RA by 31% (thus no statistical significance was observed), whereas in the control group without PVAT aliquot, the artery relaxed by only 6%.

Two way repeated measures ANOVA showed significant impact of PVAT aliquot on vessel relaxation, however the test didn’t confirm any influence of L-NMMA on neither the vessel wall tension, nor relaxation induced by PVAT aliquot.

1. Indomethacine:

n=5

|  | **INDO (-)** | **INDO (+)** | **p** |
| --- | --- | --- | --- |
| **PVAT (+)** | 23 ± 8,3% | 15 ± 3,8% | INDO (-) vs INDO (+)=0,167 |
| **PVAT (-)** | 15 ± 5,4% | 4 ± 2,6% |  |
| **p** | PVAT (+) vs PVAT (-)=0,006 | | PVAT (+)*INDO(+)=0,545 |

The serotonin elicited a contraction of the RA 28,3±7,3mN in the study group vs 25,3±8,4mN in the control group. The addition of serotonin in the presence of indomethacine had no significant impact on the contractory response in comparison to specimens subjected to serotonin itself (24,8±3,9mN vs. 28,3±7,3mN, p=NS in the study group; 26,5±7,4mN vs. 25,3±8,4mN, p=NS in the control group).

The addition of 5 ml of PVAT-treated solution relaxed RA by 23% in the study group. While in the control group treated with Krebs-Henseleit solution instead of PVAT aliquot, the relaxation was only 15%.

The addition of 5 ml of PVAT-treated solution in the presence of indomethacine relaxed RA by 15% (thus no statistical significance was observed), whereas in the control group without PVAT aliquot, the artery relaxed by only 4%.

Two way repeated measures ANOVA showed significant impact of PVAT aliquot on vessel relaxation, however the test didn’t confirm any influence of indomethacine on neither the vessel wall tension, nor relaxation induced by PVAT aliquot.

**Experiment 4:**

1. Barium chloride

n=8

|  | **BaCl_2_ (-)** | **BaCl_2_ (+)** | **p** |
| --- | --- | --- | --- |
| **PVAT (+)** | 33 ± 5,6% | 15 ± 6,6% | BaCl_2_ (-) vs BaCl_2_ (+)=0,006 |
| **PVAT (-)** | 15 ± 2,5% | -1 ± 3,8% |  |
| **p** | PVAT (+) vs PVAT (-) = 0,006 | | PVAT (+)*BaCl_2_ (+)=0,417 |

The serotonin elicited a contraction of the RA 29,8±6,5mN in the study group vs 17,3±4,9mN in the control group. The addition of serotonin in the presence of barium chloride induced higher contractory response in comparison to specimens subjected to serotonin itself (increase from 29,8±6,5mN to 49±9,7mN, p=0,007 in the study group; increase from 17,3±4,9mN to 48,7±8,7mN, p=0,006 in the control group).

The addition of 5 ml of PVAT-treated solution relaxed RA by 33% in the study group. While in the control group treated with DMSO solution instead of PVAT aliquot, the relaxation was only 15%.

The addition of 5 ml of PVAT-treated solution in the presence of barium chloride relaxed RA by 15% (thus no statistical significance was observed), whereas in the control group without PVAT aliquot, the artery relaxed by only 1%.

Two way repeated measures ANOVA showed significant impact of PVAT aliquot on vessel relaxation, also significant impact on the vessel wall tension, however the test didn’t confirm any influence of barium chloride on relaxation induced by PVAT aliquot.

1. Tetraethylamonnium

n=8

|  | **TEA (-)** | **TEA (+)** | **p** |
| --- | --- | --- | --- |
| **PVAT (+)** | 15 ± 2,5% | 11 ± 3,5% | TEA (-) vs TEA (+)=0,396 |
| **PVAT (-)** | 2 ± 2,1% | 1 ± 5,3% |  |
| **p** | PVAT (+) vs PVAT (-) = 0,015 | | PVAT (+)*TEA (+)=0,720 |

The serotonin elicited a contraction of the RA 40,3±8,1mN in the study group vs 35,4±11mN in the control group. The addition of serotonin in the presence of tetraethylamonium had no significant impact on the contractory response in comparison to specimens subjected to serotonin itself (56,3±11,8mN vs. 40,3±8,1mN, p=NS in the study group; 32,6±6,8mN vs. 35,4±11mN, p=NS in the control group.

The addition of 5 ml of PVAT-treated solution relaxed RA by 15% in the study group. While in the control group treated with Krebs-Henseleit solution instead of PVAT aliquot, the relaxation was only 2%.

The addition of 5 ml of PVAT-treated solution in the presence of TEA relaxed RA by 11% (thus no statistical significance was observed), whereas in the control group without PVAT aliquot, the artery relaxed by only 1%.

Two way repeated measures ANOVA showed significant impact of PVAT aliquot on vessel relaxation, however the test didn’t confirm any influence of TEA on neither the vessel wall tension, nor relaxation induced by PVAT aliquot.

1. 4-aminopyridine (1mM)

n=8

|  | **4-AP (-)** | **4-AP (+)** | **p** |
| --- | --- | --- | --- |
| **PVAT (+)** | 20± 6,8% | 17 ± 3,1% | 4-AP (-) vs 4-AP (+) = 0,154 |
| **PVAT (-)** | 5 ± 1,6% | -2 ± 1,6% |  |
| **p** | PVAT (+) vs PVAT (-) = 0,002 | | PVAT (+)*4-AP (+) = 0,478 |

The serotonin elicited a contraction of the RA 38,4±8,2mN in the study group vs 23,8±1,6mN in the control group. The addition of serotonin in the presence of 4-AP had no significant impact on the contractory response in comparison to specimens subjected to serotonin itself (31,6±1,7mN vs. 38,4±8,2mN, p=NS in the study group; 24,5±0,8mN vs. 23,8±1,6mN, p=NS in the control group).

The addition of 5 ml of PVAT-treated solution relaxed RA by 20% in the study group. While in the control group treated with Krebs-Henseleit solution instead of PVAT aliquot, the relaxation was only 5%.

The addition of 5 ml of PVAT-treated solution in the presence of 4-AP relaxed RA by 17% (thus no statistical significance was observed), whereas in the control group without PVAT aliquot, the artery relaxed by only 1%.

Two way repeated measures ANOVA showed significant impact of PVAT aliquot on vessel relaxation, however the test didn’t confirm any influence of 4-AP on neither the vessel wall tension, nor relaxation induced by PVAT aliquot.

1. 4-aminopyridine (5mM)

n=8

|  | **4-AP (-)** | **4-AP (+)** | **p** |
| --- | --- | --- | --- |
| **PVAT (+)** | 28 ± 3,4% | 35 ± 8,5% | 4-AP (-) vs 4-AP (+) = 0,339 |
| **PVAT (-)** | 9 ± 1,8% | 11 ± 6,2% |  |
| **p** | PVAT (+) vs PVAT (-) = 0,006 | | PVAT (+)*4-AP (+) = 0,669 |

The serotonin elicited a contraction of the RA 30±2,4mN in the study group vs 29,6±2,7mN in the control group. The addition of serotonin in the presence of 4-AP had no significant impact on the contractory response in comparison to specimens subjected to serotonin itself (44,5±8,9mN vs. 30±2,4mN, p=NS in the study group; 32,2±4,2mN vs. 29,6±2,7mN, p=NS in the control group).

The addition of 5 ml of PVAT-treated solution relaxed RA by 28% in the study group. While in the control group treated with Krebs-Henseleit solution instead of PVAT aliquot, the relaxation was only 9%.

The addition of 5 ml of PVAT-treated solution in the presence of 4-AP relaxed RA by 35% (thus no statistical significance was observed), whereas in the control group without PVAT aliquot, the artery relaxed by only 11%.

Two way repeated measures ANOVA showed significant impact of PVAT aliquot on vessel relaxation, however the test didn’t confirm any influence of 4-AP on neither the vessel wall tension, nor relaxation induced by PVAT aliquot.

1. Apamine

n=8

|  | **Apa (-)** | **Apa (+)** | **p** |
| --- | --- | --- | --- |
| **PVAT (+)** | 22 ± 4,7% | 27 ± 8,3% | Apa (-) vs Apa (+)=0,988 |
| **PVAT (-)** | 6 ± 3,3% | 1,5 ± 8,1% |  |
| **p** | PVAT (+) vs PVAT (-) = 0,016 | | PVAT (+)*Apa(+)=0,373 |

The serotonin elicited a contraction of the RA 28,6±6,2mN in the study group vs 27±6,7mN in the control group. The addition of serotonin in the presence of apamine had no significant impact on the contractory response in comparison to specimens subjected to serotonin itself (34,7±6,4mN vs. 28,6±6,2mN, p=NS in the study group; 30,1±4,2mN vs. 27±6,7mN, p=NS in the control group).

The addition of 5 ml of PVAT-treated solution relaxed RA by 22% in the study group. While in the control group treated with Krebs-Henseleit solution instead of PVAT aliquot, the relaxation was only 6%.

The addition of 5 ml of PVAT-treated solution in the presence of apamine relaxed RA by 27% (thus no statistical significance was observed), whereas in the control group without PVAT aliquot, the artery relaxed by only 1,5%.

Two way repeated measures ANOVA showed significant impact of PVAT aliquot on vessel relaxation, however the test didn’t confirm any influence of apamine on neither the vessel wall tension, nor relaxation induced by PVAT aliquot.

1. Iberiotoxin

n=8

|  | **IBX (-)** | **IBX (+)** | **p** |
| --- | --- | --- | --- |
| **PVAT (+)** | 22 ± 4,1% | 21 ± 3,4% | IBX (-) vs IBX (+)=0,654 |
| **PVAT (-)** | 4 ± 7,2% | 2 ± 3,4% |  |
| **p** | PVAT (+) vs PVAT (-) = 0,004 | | PVAT (+)*IBX (+)=0,946 |

The serotonin elicited a contraction of the RA 22,4±2,7mN in the study group vs 21,2±4,33mN in the control group. The addition of serotonin in the presence of indomethacine had no significant impact on the contractory response in comparison to specimens subjected to serotonin itself (23,7±2,7mN vs. 22,4±2,7mN, p=NS in the study group; 41,6±7,4mN vs. 21,2±4,33mN, p=NS in the control group).

The addition of 5 ml of PVAT-treated solution relaxed RA by 22% in the study group. While in the control group treated with Krebs-Henseleit solution instead of PVAT aliquot, the relaxation was only 4%.

The addition of 5 ml of PVAT-treated solution in the presence of iberiotoxin relaxed RA by 21% (thus no statistical significance was observed), whereas in the control group without PVAT aliquot, the artery relaxed by only 2%.

Two way repeated measures ANOVA showed significant impact of PVAT aliquot on vessel relaxation, however the test didn’t confirm any influence of iberiotoxin on neither the vessel wall tension, nor relaxation induced by PVAT aliquot.

1. Glibenclamide

n=8

The serotonin elicited a contraction of the RA 21,9±2,7mN in the study group vs 20,7±4,3mN in the control group.

The addition of 5 ml of PVAT-treated solution relaxed RA by 23% in the study group. While in the control group treated with DMSO solution instead of PVAT aliquot, the relaxation was only 5%.

Two way repeated measures ANOVA showed significant impact of PVAT aliquot on vessel relaxation (p=0,007).

We weren’t able to complete the experiment with glibenclamide. Despite out efforts to change the dose, we always obtained lack of any RA activity.
